# Complete Genome Sequences of Two Sweet Potato Chlorotic Stunt Virus Isolates from China

**DOI:** 10.1128/genomeA.00218-13

**Published:** 2013-05-09

**Authors:** Yanhong Qin, Zhenchen Zhang, Qi Qiao, Desheng Zhang, Yuting Tian, Yongjiang Wang, Shuang Wang

**Affiliations:** Institute of Plant Protection, Henan Academy of Agricultural Sciences, Zhengzhou, China; Henan Key Laboratory of Crop Pest Control, Zhengzhou, China; IPM Key Laboratory in Southern Part of North China for Ministry of Agriculture, Zhengzhou, China

## Abstract

Sweet potato chlorotic stunt virus (SPCSV) was first detected in China in 2010, and several partial sequences have been determined for Chinese SPCSV isolates. This report describes the complete genome sequences of two SPCSV isolates from the Guangdong and Jiangsu provinces and will be valuable for understanding the characteristics of SPCSVs in China.

## GENOME ANNOUNCEMENT

Sweet potato chlorotic stunt virus (SPCSV) is a whitefly-transmitted member of the genus *Crinivirus*, family *Closteroviridae* (1). It has a bipartite, single-stranded, plus-sense RNA genome, the second largest plant virus genome after that of the closterovirus citrus tristeza virus (2). Coinfection of sweet potato with SPCSV and sweet potato feathery mottle virus (SPFMV) causes the syndrome sweet potato virus disease (SPVD), which is characterized by general chlorosis, stunting, leaf crinkling, and even plant death (3). SPCSVs can be divided into East African (EA) and West African (WA) strains based on serology and nucleotide sequence data (4). Genome sequences for both strains have been reported for isolates from Uganda, Peru, and Spain (GenBank accession no. FJ807784 and FJ807785) (5, 6). SPCSV was recently reported in China, where it is becoming a serious problem (7, 8). Presently, only partial SPCSV sequences have been determined in China (7, 8).

We used the SPCSV RNA1 and RNA2 sequences from isolates m2-47 (Peru; GenBank accession no. HQ291259 and HQ291260) and Can181-9 (WA; GenBank accession no. FJ807784 and FJ807785) to design primer pairs to amplify four overlapping cDNA fragments covering the genomes of two SPCSV isolates from the Guangdong and Jiangsu provinces of China. RNA was extracted from infected whiteflies, and reverse transcriptase PCR (RT-PCR) was performed using these primers. Rapid amplification of cDNA ends (RACE; TaKaRa, Dalian, China) was used to capture the 5′ and 3′ ends of the viral genomes. PCR products were gel purified prior to sequencing. Genomes were assembled with DNAMAN (version 6.0). Jiangsu SPCSV isolate RNA1 is 8,637 nucleotides (nt) long, including an 89-nt 5′ untranslated region (5′-UTR) and a 193-nt 3′-UTR. RNA2 from this isolate is 8,107 nt long, including a 191-nt 5′-UTR and a 192-nt 3′-UTR. Like that from the Can181-9 isolate, RNA1 from the Jiangsu isolate contains four open reading frames (ORFs), nucleotides 90 to 6053 (polyprotein 1a), 6052 to 7569 (RdRp), 7583 to 8272 (RNase 3), and 8277 to 8444 (p7). Segment RNA2 contains nine ORFs, nucleotides 192 to 329 (p5.2), 333 to 467 (p5), 406 to 534 (p5.1), 879 to 2543 (heat shock protein 70h), 2565 to 4121 (p60), 4103 to 4324 (p8), 4352 to 5125 (major coat protein), 5128 to 7182 (minor coat protein), and 7187 to 7915 (p28). RNA1 from the Guangdong SPCSV isolate is 8,622 nt long, including an 89-nt 5′-UTR and a 172-nt 3′-UTR. RNA2 from this isolate is 8,217 nt long, including an 88-nt 5′-UTR and a 192-nt 3′-UTR. Like the EA strain m2-47 isolate, RNA1 lacks the *p22* gene and contains four ORFs, nucleotides 90 to 6053 (p227), 6052 to 7569 (RdRp), 7586 to 8272 (RNase 3), and 8277 to 8450 (p7). RNA2 contains eight ORFs, nucleotides 89 to 238 (p6), 554 to 712 (p6), 984 to 2648 (hsp70h), 2670 to 4226 (p60), 4208 to 4429 (p8), 4462 to 5235 (major coat protein), 5238 to 7292 (minor coat protein), and 7297 to 8025 (p28).

The Jiangsu RNA1 and RNA2 sequences were 98.90% and 98.68% identical, respectively, to those of the WA strain Can181-9 isolate. RNA1 and RNA2 from the Guangdong isolate were 99.27% and 99.68% identical to those from the EA strain m2-47 isolate. We present the first complete genome sequences of SPCSV EA and WA isolates from China and show that SPCSV isolates display a high degree of sequence conservation. In addition, this is the first report of the complete genome sequences of SPCSV from whitefly vectors. These data provide important insights into the genomic variation of geographical SPCSV isolates.

### Nucleotide sequence accession numbers.

The Chinese SPCSV sequences were deposited with the GenBank accession numbers KC146840 (Jiangsu RNA1), KC146841 (Jiangsu RNA2), KC146842 (Guangdong RNA1), and KC146843 (Guangdong RNA2).
